# CAREGIVER-SPECIFIC QUALITY MEASURES FOR HCBS: STAKEHOLDER PRIORITIES AND ENVIRONMENTAL SCAN

**DOI:** 10.1093/geroni/igac059.172

**Published:** 2022-12-20

**Authors:** Lauren Penney, Erin Finley, Julie Parish Johnson, Jacqueline Pugh, Luci Leykum, Polly Noel

**Affiliations:** South Texas Veterans Health Care System, San Antonio, Texas, United States; VA Greater Los Angeles Healthcare System, Los Angeles, California, United States; South Texas Veterans Health Care System, San Antonio, Texas, United States; South Texas Veterans Health Care system, San Antonio, Texas, United States; South Texas Veterans Health Care System, San Antonio, Texas, United States; South Texas Veterans Health Care System, San Antonio, Texas, United States

## Abstract

Although informal family caregivers are increasingly recognized for their essential role in helping older and/or medically-complex adults live in the community for as long as possible, their priorities and perspectives have not been well-integrated into assessments of home- and community-based services (HCBS). Our aim was to identify measurement gaps to guide quality monitoring and improve HCBS. Caregiver concerns and quality measurement priorities were identified during a multi-level stakeholder engagement process (34 Veterans, 24 caregivers, and 39 facility leaders, clinicians, and staff) across four VA healthcare systems. We conducted an environmental scan and scoping review of national quality measure sets for HCBS, comparing caregiver-specific items against stakeholder-identified concerns and priorities. Only five of eleven non-VA measure sets and three of four VA measure sets included caregiver-specific items; these did not encompass the full range stakeholder-identified concerns and priorities. Measures that emphasize caregivers can help healthcare systems monitor and improve HCBS quality.

